# Predictive classification models and targets identification for betulin derivatives as *Leishmania donovani* inhibitors

**DOI:** 10.1186/s13321-018-0291-x

**Published:** 2018-08-17

**Authors:** Yuezhou Zhang, Henri Xhaard, Leo Ghemtio

**Affiliations:** 1 Centre for Drug Research, Division of Pharmaceutical Biosciences, University of Helsinki, Viikinkaari 5E, P.O. Box 56, 00790 Helsinki, Finland; 2 Faculty of Pharmacy, Division of Pharmaceutical Chemistry and Technology, University of Helsinki, Viikinkaari 5E, P.O. Box 56, 00790 Helsinki, Finland

**Keywords:** *Leishmania donovani* inhibitors, Betulin derivatives, Predictive modeling, Classification models, Recursive partitioning, In silico target prediction, Structure-based pharmacophore, Network analysis

## Abstract

Betulin derivatives have been proven effective in vitro against *Leishmania donovani* amastigotes, which cause visceral leishmaniasis. Identifying the molecular targets and molecular mechanisms underlying their action is a currently an unmet challenge. In the present study, we tackle this problem using computational methods to establish properties essential for activity as well as to screen betulin derivatives against potential targets. Recursive partitioning classification methods were explored to develop predictive models for 58 diverse betulin derivatives inhibitors of *L. donovani* amastigotes. The established models were validated on a testing set, showing excellent performance. Molecular fingerprints FCFP_6 and ALogP were extracted as the physicochemical properties most extensively involved in separating inhibitors from non-inhibitors. The potential targets of betulin derivatives inhibitors were predicted by in silico target fishing using structure-based pharmacophore searching and compound-pharmacophore-target-pathway network analysis, first on PDB and then among *L. donovani* homologs using a PSI-BLAST search. The essential identified proteins are all related to protein kinase family. Previous research already suggested members of the cyclin-dependent kinase family and MAP kinases as Leishmania potential drug targets. The PSI-BLAST search suggests two *L. donovani* proteins to be especially attractive as putative betulin target, heat shock protein 83 and membrane transporter D1.

**Electronic supplementary material:**

The online version of this article (10.1186/s13321-018-0291-x) contains supplementary material, which is available to authorized users.

## Background

Leishmaniasis is a neglected tropical disease caused by Leishmania protozoan parasites that affect millions of people worldwide [1–3]. During the past decade, leishmaniasis has spread considerably, and an increasing number of new cases are being reported every year [3]. Several treatments exist for leishmaniasis [4], but they are not fully active, have adverse effects, loss of efficacy and are highly expensive [5]. Hence, there is an urgent need to develop new, safe and effective medications.

Betulin derivatives have a significant in vitro inhibition growth of *L. donovani* amastigotes, which cause visceral leishmaniasis, the most severe form of the disease [6, 7]. Betulinic acid and other betulin derivatives have furthermore remarkable antiviral [8–11], anti-HIV [12], antiulcer [13], anti-inflammatory [14, 15], anti-malaria [16, 17] and anti-tumoral [18–20] activity that make this class of compounds promising for new drugs discovery [21–24]. Structure–activity relationships and pharmacological properties of betulin have been studied previously [25–29]. Recently, our collaborators have synthesized 58 betulin heterocyclic derivatives and evaluated their activity and selectivity against *L. donovani* amastigotes with similar or better inhibitory activity (> 80%) than some well-known antibiotics (Nystatin, Pentamycin, Amphotericin) [6, 30, 31]. Computational methods such as QSAR [32] and pharmacophore modeling [33] are important methods in modern drug discovery that have been successfully applied for modeling activities of betulin derivatives [34–42]. However, the congeneric series are still limited, and the mechanism of action of these compounds are still undefined. To date, very few computational studies and models have been done on Betulin derivatives to explore the full potential of this class of compounds, with one derivatives in clinical phase 3 (Oleogel-S10), and accelerate the understanding of their mode of action. In the present study, we report an application of classification method, recursive partitioning (RP) to build predictive models of the inhibitory activity of betulin derivatives and characterize their molecular properties. RP models can select essential molecular descriptors according to the decrease of the performance resulting from the random permutation of the variables. Also, we investigated the compound-target interaction network and potential pharmacological actions by reverse pharmacophore database screening. Although it can be to some extent debated [43], it is commonly accepted that structurally similar compounds have similar biological activity [44] and may also recognize homologous targets across organisms [45]. This concept spurs us to assume the proteins interacting with compounds that are similar to betulin derivatives in the structure are potential binding targets as well. We thus screened potent betulin inhibitors of Leishmania growth against PharmaDB [46], a database containing a collection of pharmacophores model built from protein-ligand complexes, to identify possible targets.

## Materials and methods

### Compounds and biological data

The molecular structures and biological data used in this study, 58 betulin derivatives synthesized by the Yli-Kauhaluoma group, were retrieved from references [6, 30, 31] (Table 1). The biological activities are reported as the percentage inhibition of *L. donovani* axenic amastigotes growth at 50 μM concentrations. Three datasets were generated, and the compounds were categorized in different classes depending on their % of inhibition (%I) in three different ways (Table 2). Dataset 1, the compounds were divided into two classes as active (%I ≥ 49) and inactive (%I < 49). Dataset 2, the compounds were divided into three classes as active (%I > 69), moderate active (%I ≥ 36 et ≤ 69) and inactive (%I < 36). Dataset 3, is similar to Dataset 2 but the group of moderately active compounds, considered as an uncertainty buffer, is not used.

**Table 1:**
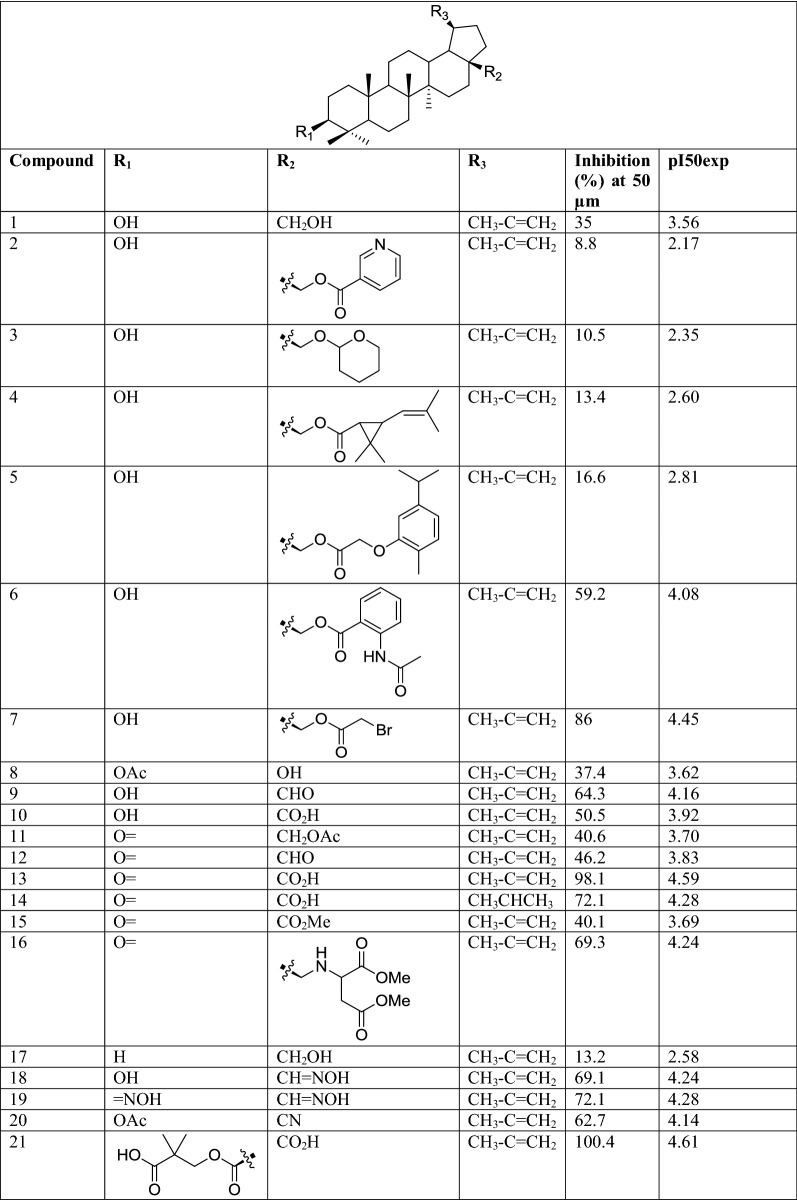

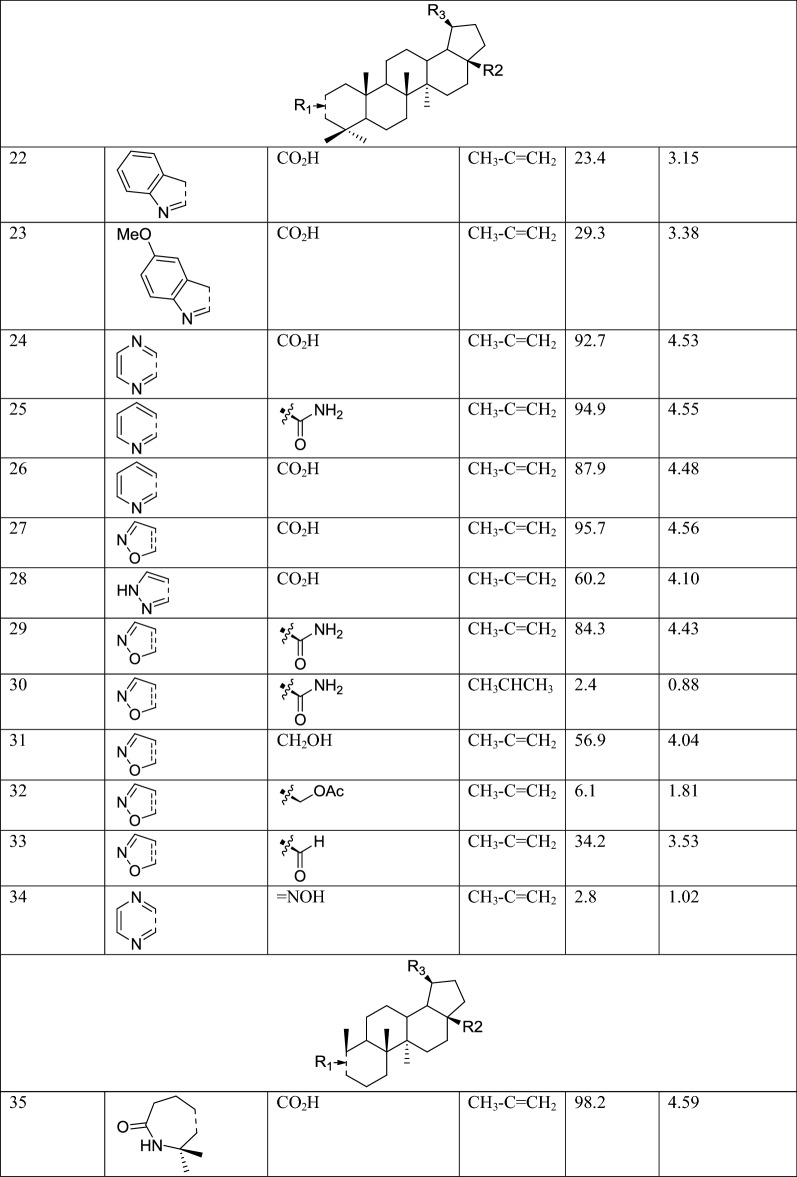

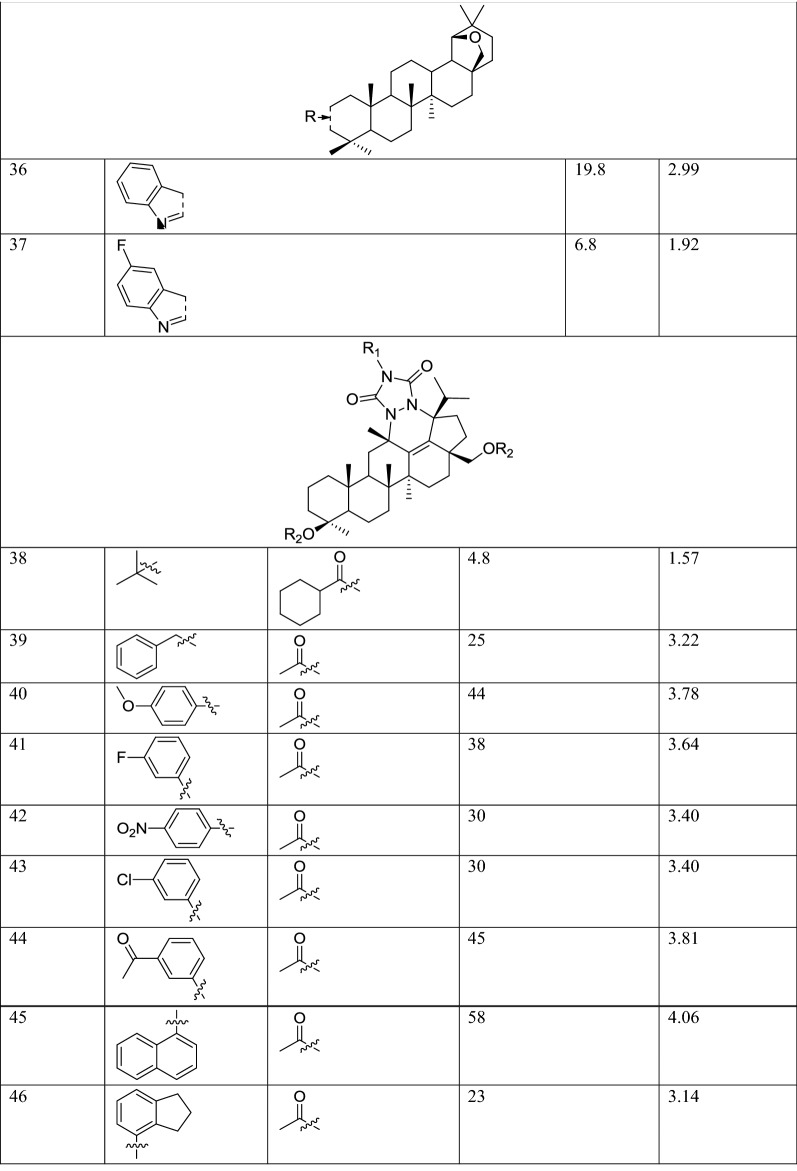

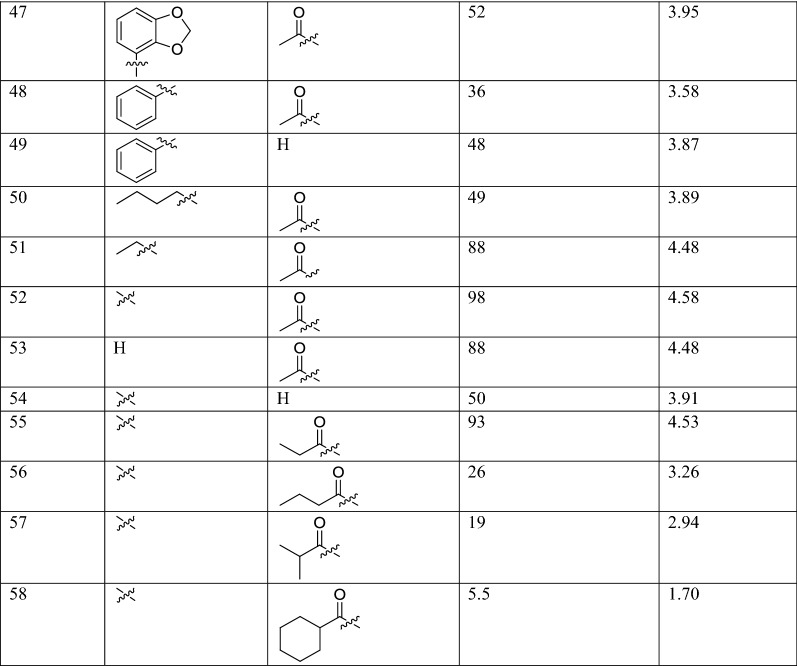
Experimental Leishmanial growth inhibitory activities of betulin derivatives against *L. donovani* axenic amastigotes

**Table 2:** Dataset used for recursive partitioning classification models

	Class 1 (inactive)	Class 2 (moderate active)	Class 3 (active)	Total
Dataset 1	Activity < 49		Activity ≥ 49	
	31	0	27	58
Dataset 2	Activity < 36	36 ≥ Activity ≤ 69	Activity > 69	
	22	19	17	58
Dataset 3	Activity < 36	36 ≥ Activity ≤ 69	Activity > 69	
	22	Excluded	17	39

### Generating the molecular structures and conformational analysis

The skeleton of betulin derivatives was drawn using ChemBioDraw Ultra 12.0, assigning hydrogen atoms with Maestro 9.6 (Schrödinger). After that, the dataset was prepared by Discovery Studio 4.5 (Accelrys Inc.) (DS 4.5). Partial charges of structures were calculated based on the CHARMm force field. Full minimization was run with the Smart minimizer algorithm until root mean square gradient was 0.01 and maximum 2000 steps. No implicit solvent model was included.

### Recursive partitioning (RP) models

RP is a classification method for multivariable data analysis. It creates a decision tree to correctly classify and uncover relationships between members of the dataset based on a dichotomous splitting of a dependent property, in our case compounds properties and their %I. RP analysis was carried out using DS 4.5 to develop decision trees that categorize the compounds into two and three classes based on the % inhibition. RP single tree (ST) models and multi-tree bagged forest (BF) models made up of multiple trees were used. Both ST and BF models are particularly appropriate in case of imbalanced training data and are easily interpretable, while also providing a significant degree of predictive accuracy [47–50]. For both methods, a training set was used to build the decision trees, and a test set was utilized to evaluate the predictive power of the models. Using two splitting methods, we generated two training and test sets from each of the three datasets (see Tables 3 and 4). The first method (split by diversity) assigns a diverse subset of compounds to the training and test set. The second way (random per cluster) cluster the compounds by similarity and then randomly assigned compounds from each group between the training and test set. Both methods use 2D fingerprint molecular descriptors and a proportion of 70% data for the training versus 30% for the test set.

**Table 3:** Bagged forest models

	Splitting method	Training			Test	
		Dataset	ROC score		Dataset	ROC score
			In-bag training	Out-of-bag training		
Dataset 1 (2 Class)	Diversity	1 (20) 2 (21)	0.97	0.72	1 (11) 2 (6)	0.73
	R C	1 (25) 2 (17)	0.98	0.63	1 (6) 2 (10)	0.73
Dataset 2 (2 Class from 3)	Diversity	1 (13) 2 (13)	0.99	0.59	1 (9) 2 (4)	0.87
	R C	1 (20) 2 (9)	0.97	0.71	1 (2) 2 (8)	0.94
Dataset 3 (3 Class)	Diversity	1 (13) 2 (15) 3 (13)	0.96	0.58	1 (8) 2 (4) 3 (4)	0.67
	R C	1 (20) 2 (13) 3 (9)	0.97	0.65	1 (2) 2 (6) 3 (8)	0.59

**Table 4:** Simple tree models

			Training			Test
		Dataset	ROC score	Roc score (cross-validated)	Dataset	ROC score
Dataset 1 (2 Class)	Diverse	1 (20) 2 (21)	0.89	0.62	1 (11) 2 (6)	0.71
	R C	1 (25) 2 (17)	0.91	0.62	1 (6) 2 (10)	0.91
Dataset 2 (2 Class from 3)	Diverse	1 (13) 2 (13)	0.91	0.63	1 (9) 2 (4)	0.80
	R C	1 (20) 3 (9)	0.94	0.57	1 (2) 3 (8)	0.59
Dattaset 3 (3 Class)	Diverse	1 (13) 2 (15) 3 (13)	0.90	0.65	1 (8) 2 (4) 3 (4)	0.70
	R C	1 (20) 2 (13) 3 (9)	0.76	0.54	1 (2) 2 (6) 3 (8)	0.72

BF has a relatively small number of trees (10) generated using a separate bootstrap sample of the original data for each tree. All descriptors are considered as possible splitting criteria for each node and weighting method is set to “by class” by default, to compensate for imbalanced data. All others parameters were set to default. BF can measure how each descriptor contributes to the prediction accuracy in the course of training. We estimated the predictive ability of the ST models with five fold cross-validation and BF models using out-of bag statistics. For BF, in each bootstrap training set, around one-third of the instances are left out, constituting the out-of-bag sample. The test set was used to estimate the fitting ability of the ST and RF models on a new dataset that was not used in the model construction. The performance of the ST and BF models are based on three metrics: true positive rate (recall or sensitivity), specificity, and the area under the curve (AUC) of the receiver operating characteristics (ROC) plot. AUC or ROC score represents the probability that a classifier will be estimated correctly, with values 0.5 indicating better than random prediction and 1 signifying perfect prediction [51].

### Target fishing

By screening a compound against a panel of pharmacophore models derived from multiple pharmacological targets, the potential targets of the compound can be outlined. Automated ligand profiling available in DS 4.5 so-called “Ligand Profiler” protocol was used [52]. DS 4.5 is equipped with a pharmacophore database PharmaDB that is the largest ever-reported collection of structure-based pharmacophores, 68,056 entries from 8166 protein-ligand X-ray structures [46, 53, 54]. These pharmacophores are derived from the sc-PDB dataset, a collection of 3D structures of binding sites found in the Protein Data Bank. For most actives betulin derivatives, all the pharmacophore models from PharmaDB were selected for the virtual screening with default settings. The rigid mode was used as the molecular mapping algorithm. No molecular features were allowed to be missed while mapping these compounds to the pharmacophore models to increase selectivity. The minimal inter-feature distance was set at 0.5 Å. For each target, the name and pathway information was collected from ChEMBL [55] and WikiPathways [56] databases using KNIME [57] version 3.1.2. Compound-Target-Pathway networks were generated by Cytoscape 3.0 (Cytoscape Consortium, USA) [58] where network nodes illustrate compounds, targets, and biological pathways. The edges linking the compound-target and target-pathway describe their relationships. Position-Specific Iterated BLAST (PSI-BLAST) search is done to identify the homologous protein in *L. donovani* from the selected target as the query sequence [59].

## Results

### Structural diversity analysis, RP (ST/BF) model development and interpretation

**Fig. 1:**
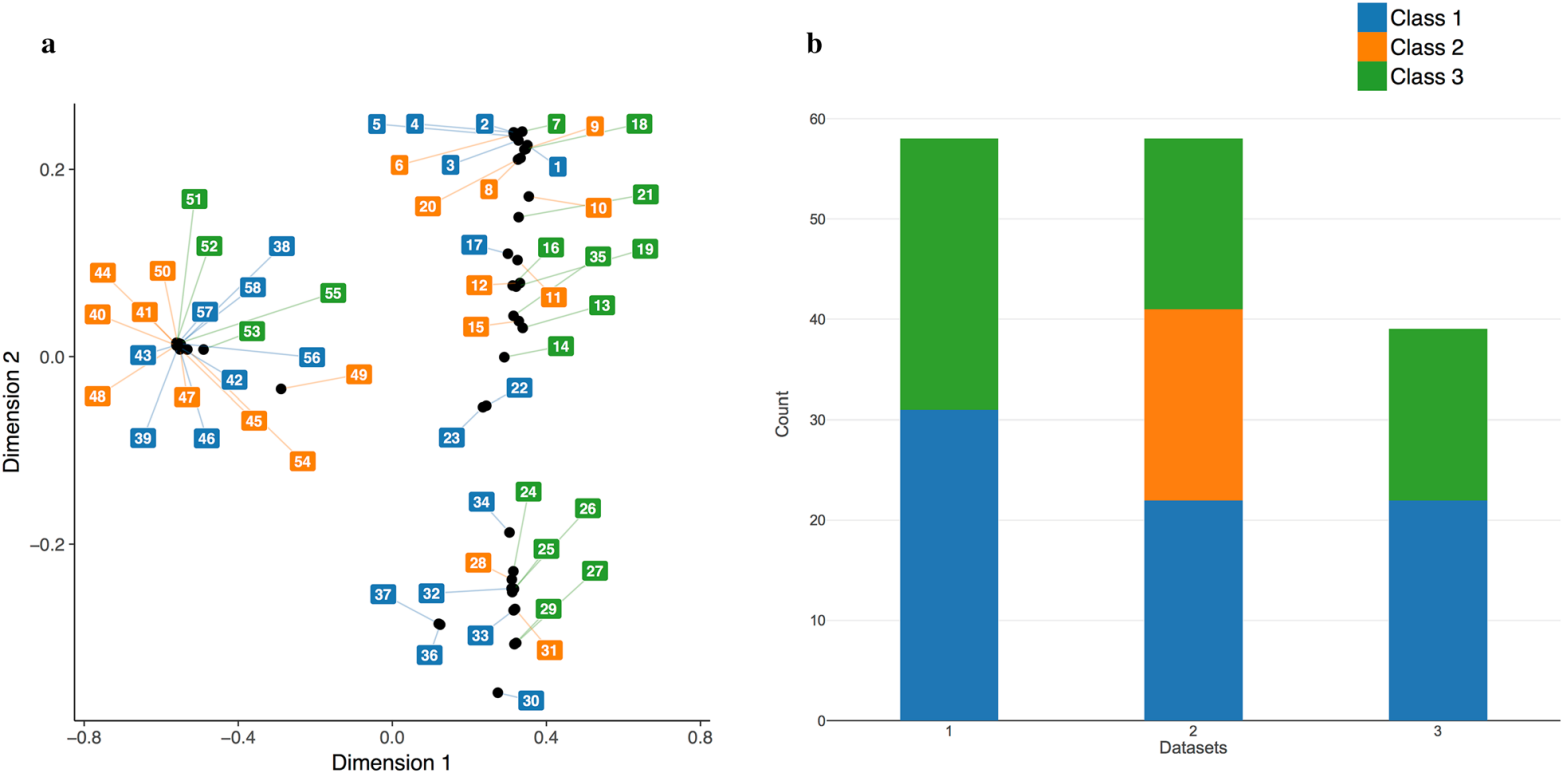
**a** Compounds similarity distance (Tanimoto score). **b** Distribution of compounds in % inhibition classes for the three datasets

The robustness and efficiency of classification models are usually affected by the diversity of dataset used for modeling, with the effect that the more diverse are the compounds, the broader will be the applicability of the model. The dissimilarity between any two molecules was computed using a Tanimoto coefficient. In this study, the average fingerprint distance for the dataset of 58 betulin derivatives inhibitors is 0.7 with a minimum of 0.12 and maximum at 0.9. Figure 1a shows a broad range of diversity across compounds. Also, the data set has an average molecular property distance of 1.33, minimum at 0.067 and maximum at 2.75 which shows good structural and property diversity of the dataset. Two different methods, diversity and random per cluster were used to split the dataset into test and training sets. (see Tables 3 and 4) Different inhibitory classes with varying distributions of training-test where thus created (Fig. 1b).

Detailed result of ST and BF models are reported Tables 3 and 4 respectively. The ST and RF models performance are comparable. As shown, BF was able to find predictive models from dataset 2 with both splitting method. The ROC score for the in-bag training data for all trees in the forest model is 0.99 and 0.96, and the out-of-bag ROC score is 0.59 and 0.71 for the training set. The in-bag results are predictions for the data used to train the tree, while the out-of-bag results are predictions for the left-out data. The external test sets including 13 and 10 compounds respectively were used to evaluate the predictive ability of the two models. The ROC score on external test sets is good, 0.87 and 0.94 respectively. The confusion matrix, as well as sensitivity and specificity values, are presented in Additional file 1: Table S1, Additional file 2: Table S2. In the betulin derivatives inhibitors models, RF and ST method can correctly classify most of the molecules of the external test set. These outcomes indicate that the developed ST and RF models show favorable and robust prediction performance. The Y-randomization test was performed four times, and the AUC values for the model using the data set with experimental activity values were significantly higher than those obtained from the dataset with randomized values, indicating the robustness of our models. The most suitable sets of molecular descriptors for predicting Betulin derivatives inhibitors were extracted from the RF prediction models via feature selection. A summary of descriptors based on their frequency of occurrences in the models are given in Table 5. The FCFP_6 feature, number aromatic rings, number rings, molecular fractional polar surface area, molecular weight, number rotatable bonds are predominant in all models. In general, the frequency at which a descriptor was selected empirically appears to distinguish truly important descriptors from others best. In the RF models of betulin derivatives inhibitors, FCFP_6 feature, number aromatic rings are the most critical descriptors for classification.

**Table 5:** Forest important features used to build the models

Property attributes
FCFP_6
ALogP
Molecular_Weight
Num_H_Donors
Num_H_Acceptors
Num_RotatableBonds
Num_Rings
Num_AromaticRings
Molecular_FractionalPolarSurfaceArea

### Profiling results

**Fig. 2:**
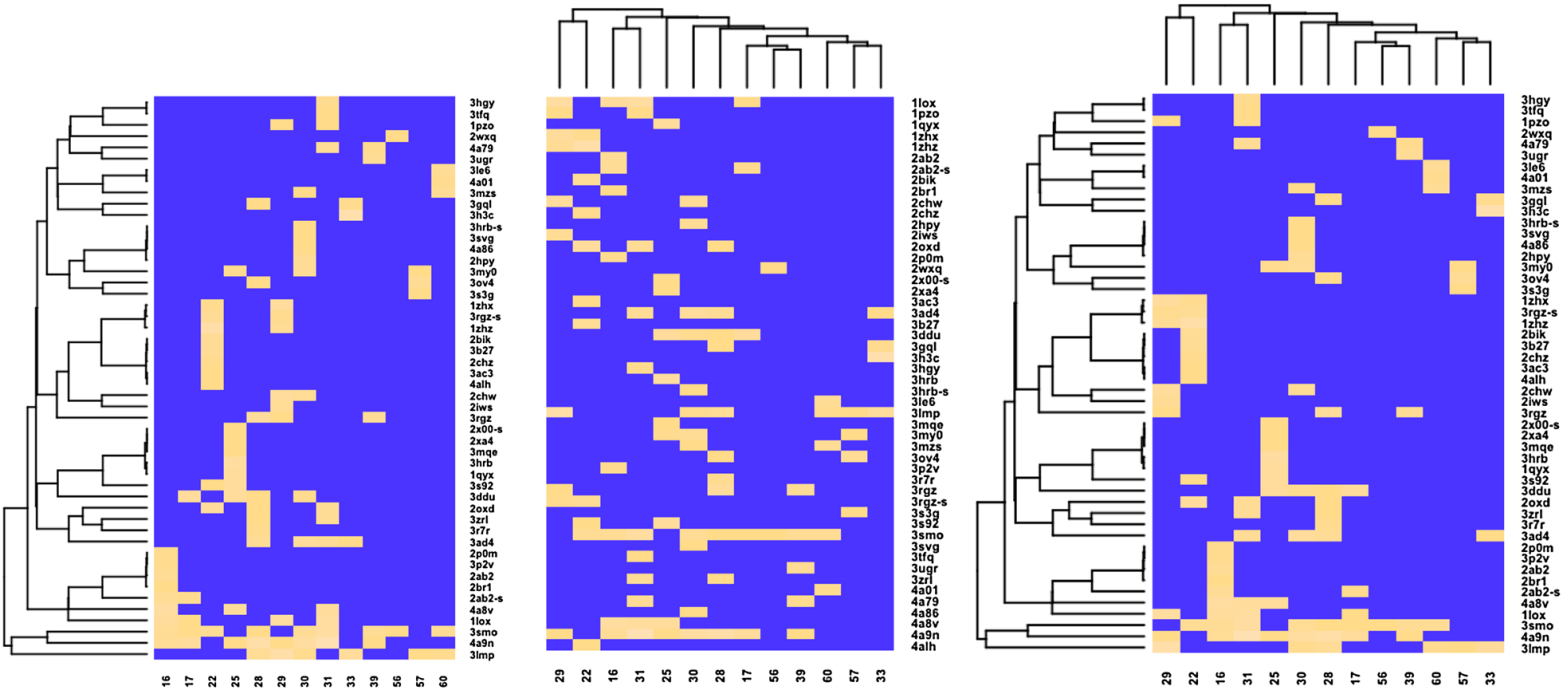
Compounds that fit each pharmacophore with shape fit value > 0.9

The profiling results from 13 most actives compounds are presented in Table 6. The fit value was used to measure the fitness of the ligand and pharmacophore. A fit value equal or higher to 0.9 was used as a threshold to select targets from the activity profiler result (see Fig. 2). The 13 compounds mapped 47 pharmacophores models out of a total of 68,056 models with a rigid mapping and the presence of all molecular features required. These models belonged to 32 protein targets and were involved in 184 pathways. Protein sequences of all the predicted targets were collected, and a blast search was run on NCBI server to identify homolog in *L. donovani* (Table 7).

**Table 6:** Targets selected by the target pharmacophore screening with fit value > 0.9

ID	Uniprot ID	Gene name	Title	Family
3ac3	P06239	LCK_HUMAN	Tyrosine-protein kinase Lck	Protein kinase
3ad4	P06239	LCK_HUMAN	Tyrosine-protein kinase Lck	Protein kinase
3mzs	P00189	CP11A_BOVIN	Cholesterol side-chain cleavage enzyme, mitochondrial	Cytochrome P450
3my0	P37023	ACVL1_HUMAN	Serine/threonine-protein kinase receptor R3	Protein kinase
1lox	P12530	LOX15_RABIT	Arachidonate 15-lipoxygenase	Lipoxygenase
1qyx	P14061	DHB1_HUMAN	Estradiol 17-beta-dehydrogenase 1	Short chain dehydrogenases/reductases (SDR)
2bik	P11309	PIM1_HUMAN	Serine/threonine-protein kinase pim-1	Protein kinase
2br1	O14757	CHK1_HUMAN	Serine/threonine-protein kinase Chk1	Protein kinase
2chw	P48736	PK3CG_HUMAN	Phosphatidylinositol 4,5-bisphosphate 3-kinase catalytic subunit gamma isoform	PI3/PI4 kinase
2chz	P48736	PK3CG_HUMAN	Phosphatidylinositol 4,5-bisphosphate 3-kinase catalytic subunit gamma isoform	PI3/PI4 kinase
2p0m	P12530	LOX15_RABIT	Arachidonate 15-lipoxygenase	Lipoxygenase
2wxq	Q3UDT3	Q3UDT3_MOUSE	Phosphatidylinositol 4,5-bisphosphate 3-kinase catalytic subunit delta isoform	PI3/PI4 kinase
3ddu	P48147	PPCE_HUMAN	Prolyl endopeptidase	Peptidase S9A
3h3c	Q14289	FAK2_HUMAN	Protein-tyrosine kinase 2-beta	Protein kinase
3hrb	Q16539	MK14_HUMAN	Mitogen-activated protein kinase 14	Protein kinase
3le6	P24941	CDK2_HUMAN	Cyclin-dependent kinase 2	Protein kinase
3mqe	Q05769	PGH2_MOUSE	Prostaglandin G/H synthase 2	Prostaglandin G/H synthase
3p2v	P15121	ALDR_HUMAN	Aldose reductase	Aldo/keto reductase
3r7r	P48736	PK3CG_HUMAN	Phosphatidylinositol 4,5-bisphosphate 3-kinase catalytic subunit gamma isoform	PI3/PI4 kinase
3rgz	O22476	BRI1_ARATH	Protein brassinosteroid insensitive 1	Protein kinase
3s3g	P15121	ALDR_HUMAN	Aldose reductase	Aldo/keto reductase
3tfq	P28845	DHI1_HUMAN	Corticosteroid 11-beta-dehydrogenase isozyme 1	Short chain dehydrogenases/reductases (SDR)
3ugr	P42330	AK1C3_HUMAN	Aldo-keto reductase family 1 member C3	Aldo/keto reductase
3zrl	P49841	GSK3B_HUMAN	Glycogen synthase kinase-3 beta	Protein kinase
4a79	P27338	AOFB_HUMAN	Amine oxidase [flavin-containing] B	Flavin monoamine oxidase
2hpy	P02699	OPSD_BOVIN	Rhodopsin	G-protein coupled receptor 1
3gql	P11362	FGFR1_HUMAN	Fibroblast growth factor receptor 1	Protein kinase
2ab2	P08235	MCR_HUMAN	Mineralocorticoid receptor	Nuclear hormone receptor
3lmp	P37231	PPARG_HUMAN	Peroxisome proliferator-activated receptor gamma	Nuclear hormone receptor
1pzo	P62593	BLAT_ECOLX	Beta-lactamase TEM	Class-A beta-lactamase
1zhx	P35844	KES1_YEAST	Oxysterol-binding protein homolog 4	Oxysterol-binding protein
1zhz	P35844	KES1_YEAST	Oxysterol-binding protein homolog 4	Oxysterol-binding protein
2oxd	P28523	CSK2A_MAIZE	Casein kinase II subunit alpha	Protein kinase
2x00	Q8WSF8	Q8WSF8_APLCA	Soluble acetylcholine receptor	Neurotransmitter-gated ion-channel
3hgy	Q7B8P6	Q7B8P6_CAMJU	cmeR	Transcriptional regulator
3ov4	P00947	SDIS_COMTE	Steroid delta-isomerase	Steroid delta-5-4-isomerase
3s92	Q15059	BRD3_HUMAN	Bromodomain-containing protein 3	Bromodomain and extraterminal domain
3smo	P31947	1433S_HUMAN	14-3-3 protein sigma	14-3-3 protein
4a01	O22124	O22124_VIGRA	Pyrophosphate-energized vacuolar membrane proton pump	H(+)-translocating pyrophosphatase (TC 3.A.10)
4a86	P15494	BEV1A_BETPN	Major pollen allergen Bet v 1-A	Bet v I type allergen
4a8v	P43183	BEV1J_BETPN	Major pollen allergen Bet v 1-J	Bet v I type allergen
2iws	P02829	HSP82_YEAST	ATP-dependent molecular chaperone HSP82	Heat shock protein Hsp90
3b27	P07900	HS90A_HUMAN	Heat shock protein HSP 90-alpha	Heat shock protein Hsp90
2xa4	O60674	JAK2_HUMAN	Tyrosine-protein kinase JAK2	Protein kinase
4a9n	P25440	BRD2_HUMAN	Bromodomain-containing protein 2	Bromodomain and extraterminal domain
4alh	P25440	BRD2_HUMAN	Bromodomain-containing protein 2	Bromodomain and extraterminal domain
3svg	O60885	BRD4_HUMAN	Bromodomain-containing protein 4	Bromodomain and extraterminal domain

**Table 7:** *Leishmania donovani* homologous targets from PSIBLAST search wit evalue < 3

Uniprot ID	Gene name	Title	%Identity	Evalue	Bit score	Query
P27890	HSP83_LEIDO	Heat shock protein 83	61.76	0	568	P07900|HS90A_HUMAN
Q01440	GTR1_LEIDO	Membrane transporter D1	34.83	0.004	30.8	P21616|AVP_VIGRR
A4ZZ93	DHYSL_LEIDO	Inactive deoxyhypusine synthase	19.70	0.042	26.2	P42330|AK1C3_HUMAN
P39050	TYTR_LEIDO	Trypanothione reductase	64.71	0.051	27.7	P11362|FGFR1_HUMAN
E9BDA8	GMPR_LEIDB	GMP reductase	25.64	0.073	25.4	P24941|CDK2_HUMAN
P43151	GPA_LEIDO	Putative guanine nucleotide-binding protein subunit alpha	27.03	0.099	25.4	P49841|GSK3B_HUMAN
P17804	HSP70_LEIDO	Heat shock 70 kDa protein	33.33	0.24	23.9	P62593|BLAT_ECOLX
P36889	SAHH_LEIDO	Adenosylhomocysteinase	26.72	0.24	23.5	P28845|DHI1_HUMAN
A7LBL2	PURA_LEIDO	Adenylosuccinate synthetase	27.66	0.27	25	Q15059|BRD3_HUMAN
Q27675	CYAA_LEIDO	Receptor-type adenylate cyclase A	29.73	0.31	21.6	P00947|SDIS_COMTE
P23223	GP63_LEIDO	Leishmanolysin	37.84	0.33	24.3	P37231|PPARG_HUMAN
Q25263	CYAB_LEIDO	Receptor-type adenylate cyclase B	28.36	0.52	24.6	Q3UDT3|Q3UDT3_MOUSE
P21620	IMDH_LEIDO	Inosine-5’-monophosphate dehydrogenase	47.37	0.57	22.7	P15121|ALDR_HUMAN
Q9BIC6	PFKA_LEIDO	ATP-dependent 6-phosphofructokinase	28.95	0.59	22.7	P14061|DHB1_HUMAN
Q05889	LPG1_LEIDO	Galactofuranosyl glycosyltransferase	28.07	0.69	24.6	O60885|BRD4_HUMAN
D9IFD5	DOHH_LEIDO	Deoxyhypusine hydroxylase	38.46	0.71	23.1	P37023|ACVL1_HUMAN
Q01441	GTR2_LEIDO	Membrane transporter D2	38.46	0.79	23.1	P27338|AOFB_HUMAN
P12522	ATXB_LEIDO	Probable proton ATPase 1B	25.69	1.1	22.3	P35844|KES1_YEAST
P11718	ATXA_LEIDO	Probable proton ATPase 1A	25.23	1.2	22.3	P35844|KES1_YEAST
O00874	DPOLA_LEIDO	DNA polymerase alpha catalytic subunit	22.22	1.2	23.1	P48147|PPCE_HUMAN
P55905	COQ5_LEIDO	2-methoxy-6-polyprenyl-1,4-benzoquinol methylase, mitochondrial	41.18	1.5	22.3	P02829|HSP82_YEAST
Q05885	ARD1_LEIDO	N-terminal acetyltransferase complex ARD1 subunit homolog	32.56	1.6	21.9	P02829|HSP82_YEAST
P12522	ATXB_LEIDO	Probable proton ATPase 1B	50	1.8	21.6	P35844|KES1_YEAST
P27116	DCOR_LEIDO	Ornithine decarboxylase	29.55	2.2	22.7	P48736|PK3CG_HUMAN
Q36736	KM11_LEIDO	Kinetoplastid membrane protein 11	27.59	2.6	19.2	P31947|1433S_HUMAN
Q25264	DCAM_LEIDO	S-adenosylmethionine decarboxylase proenzyme	42.11	2.7	21.6	Q15059|BRD3_HUMAN
B5APK2	DHYS_LEIDO	Deoxyhypusine synthase	36.36	2.9	20.4	P28845|DHI1_HUMAN

### Pharmacological network analysis

**Fig. 3:**
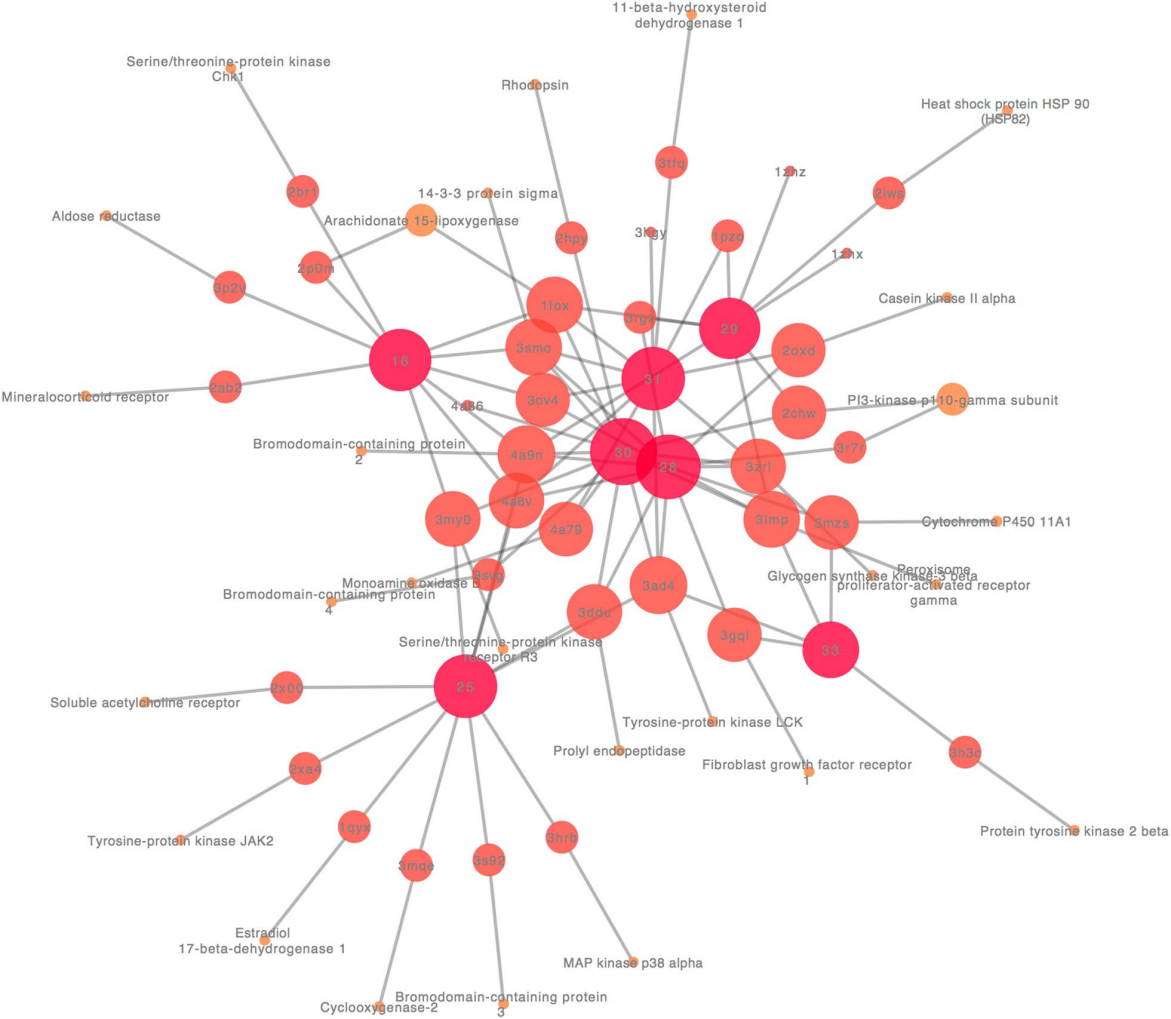
Subset of the pharmacological network of *L. donovani* Betulin derivatives inhibitors limited to the hubs of the network for target prediction. Betulin derivatives inhibitors, pharmacophore, and targets with a red to gray gradient scale

**Fig. 4:**
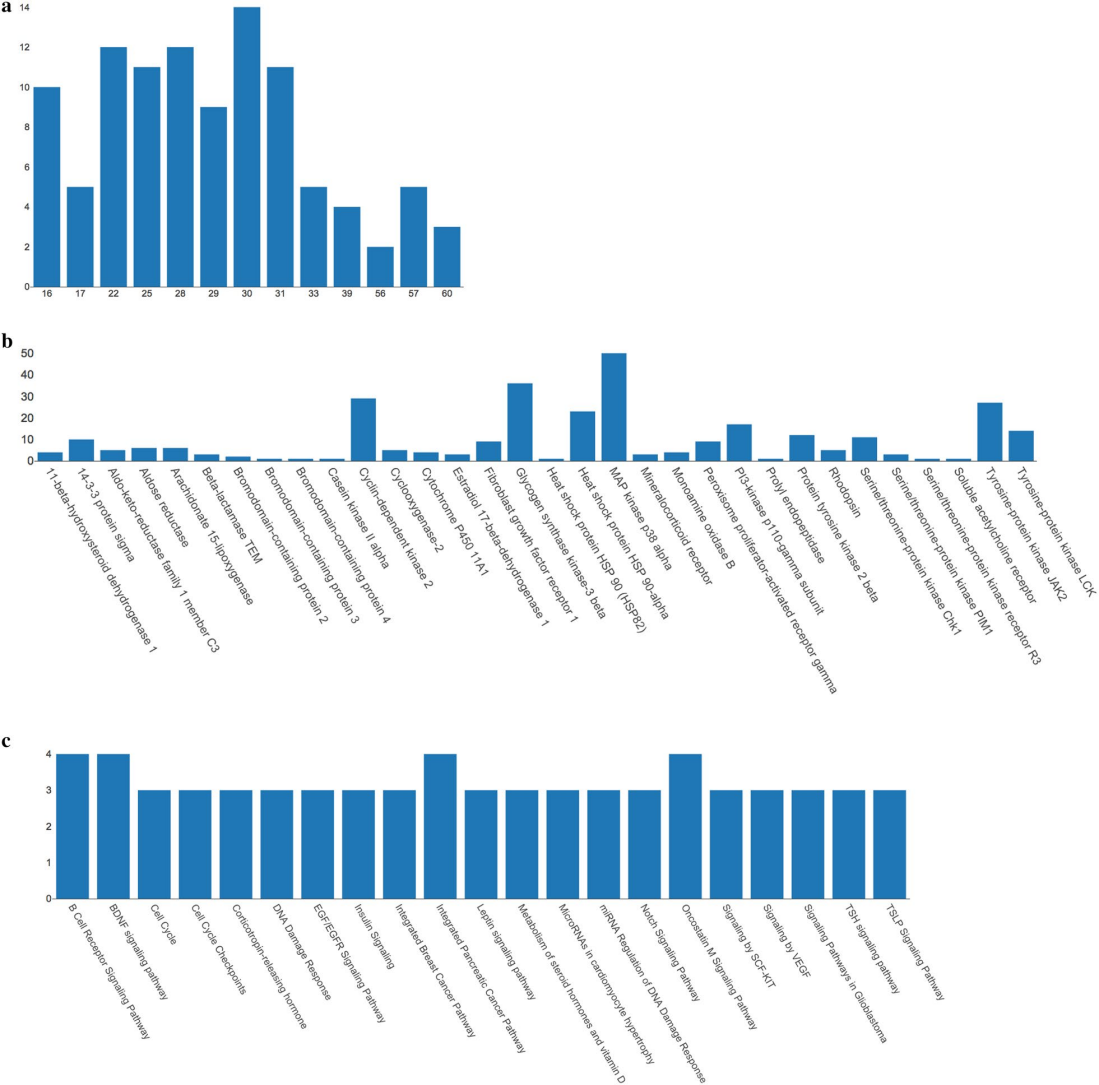
Nodes degree distribution in the pharmacological Betulin derivatives inhibitors network. **a** Degree distribution of Betulin derivatives inhibitor nodes, **b** degree distribution of target nodes, **c** degree distribution of pathway nodes

A topological analysis of the network pharmacology compound-pharmacophore-target-pathway offered insights into the biologically relevant connectivity patterns, and profoundly essential targets or pathways. A general overview of the global topological properties of the network was obtained from the statistical data by the Network Analyzer of Cytoscape. The full pharmacological network of *L. donovani* betulin derivatives inhibitors had three types of nodes, compounds, pharmacophores, and targets with related pathway information (Additional file 3: Fig. S1). The 13 compounds nodes formed the core of the network which fit 47 pharmacophores and was surrounded by the target nodes. Each target was linked to at least one pathway. A total of 209 pathway nodes constituted the outer layer of the network. Most pharmacophores were the center of a sub network-shaped connection. For seven targets, no pathway was identified. Three pharmacophores are involved in a little number of pathways, between 2 and 3 for each proposed target. Six pharmacophores formed a closed network of 2–4 pathways for each target. Pharmacophores, targets, and pathways were strongly interconnected in many-to-many relationships. Figure 3 presents a subset of the pharmacological network of *L. donovani* betulin derivatives inhibitors limited to its most connected compounds and targets nodes. The diameter of the network was 10, the centralization was 0.18, and the density was 0.011. To reduce the number of candidate targets and identify more potential targets based on targets identified from network pharmacology, the degrees distribution of all the alkaloids (Fig. 4a) and essential targets (Fig. 4b) were investigated. The compounds with higher degree values (≥ 9), such as 1, 3, 4, 5, 6, 7 and 8, that participate in more interactions than the other components are the hubs in the network. The target degree values ranged between 1 and 50. The targets with the highest degree (≥ 10) values are MAP kinase p38 alpha (50), Glycogen synthase kinase-3 beta (36), Cyclin-dependent kinase 2 (29), Tyrosine-protein kinase JAK2 (27), Heat shock protein HSP 90-alpha (23), PI3-kinase p110-gamma subunit (17), Tyrosine-protein kinase LCK (14), Tyrosine-protein kinase 2 beta (12), Serine/threonine-protein kinase Chk1(11) and 14-3-3 protein sigma (10). The highly connected nodes are referred to as the hubs of the network for target prediction. To find the relations between target proteins and the critical pathway further, we analyzed the target-pathway network. Logically, the weight of one pathway which contains many druggable target proteins is more significant than for many pathways including a single target protein that can be actioned by many drug molecules. The critical pathways (highest degree level) are summarized in Fig. 4c. These results suggested that B Cell Receptor Signaling, Brain-derived neurotrophic factor (BDNF) signaling, Integrated Pancreatic Cancer, Oncostatin M Signaling pathways may bind compounds with pharmacophoric similarities to betulin derivatives. Homologous targets were identified *in L. donovani* from the PSI-BLAST search as the potential target of Betulin derivatives. Table 7 shows a summary of *L. donovani* homologous targets with E-value < 3. A total of 27 proteins selected as similar to one or more targets identified by target fishing.

## Discussion

It is well known that the unknown targets and underlying mechanisms restrict the development of novel therapeutics against Leishmania. In silico predictive modeling offer new tools to overcome these shortages. However, many existing methodologies offers complex predictive models and relative applicability by the experimental chemist. To increase the utility, we proposed classification models and compounds-target-pathway interaction network to predict Leishmania activity of new compounds and discern the targets and potential pathways from a set of betulin derivatives active in vitro against *L. Donovani.* We successfully build two type of recursive partitioning classification models, single tree and bagged forest models. A forest model is less directly interpretable than a single-tree model in that there is not merely one tree to interpret, but depending on the type of forest, anywhere from tens to hundreds of trees. On the other hand, a forest model provides statistical measures of the relative importance of the various descriptors in distinguishing among the different classes, which is not available with single-tree models. When none of the descriptors is strongly correlated with the class membership, single-tree models can be brittle, in that a relatively small change in the training data results in a significant difference in the structure of the tree, and thus in the tree’s predictions. A forest model helps to address this problem. In principle, Network analysis has the potential to allow the target identification of *L. donovani* betulin derivatives inhibitors. The proteins in the hubs of the network (highly connected nodes) are highly associated with each other. The most critical proteins with high degree value are all related to protein kinase family. Among them, MAP kinase p38 alpha, Glycogen synthase kinase-3 beta, Cyclin-dependent kinase 2, Tyrosine-protein kinase JAK2, Heat shock protein HSP 90-alpha, PI3-kinase p110-gamma subunit, Tyrosine-protein kinase LCK, Protein tyrosine kinase 2 beta, Serine/threonine-protein kinase Chk and 14-3-3 protein sigma. They are involved in directing cellular responses to a diverse array of stimuli (such as mitogens, heat shock, and pro-inflammatory cytokines) and regulate proliferation, gene expression, mitosis, cell survival, apoptosis and many other cell functions [60]. The mode of action of these critical proteins may be done through the integrated biological network rather than by individual target. The four central pathways, B Cell Receptor, Brain-derived neurotrophic (BDNF), Integrated Pancreatic Cancer and Oncostatin M, have higher frequencies than the rest. Members of the cyclin-dependent kinase family and MAP kinases had been previously identified as essential for Leishmania and suggested as potential drug targets [61]. Homologous targets, Heat shock protein 83 and Membrane transporter D1 were identified as a possible target in *L. donovani* and proposed for experimental validation. Among the chaperones, heat shock protein 83 (Hsp83) is alternately referred to as Hsp90 or Hsp86 due to the variable molecular weight amongst different orthologues is a family of emerging targets for infectious diseases. Hsp83 is best known as cancer targets with some drug candidates in clinical development [62, 63]. Transporters are proteins that play a role in bringing small molecules across biological membranes. The function of transporters as therapeutic targets is a well-established new field of research [64]. Transporters are new therapeutic targets for treating rare diseases. But there is no, till today, a case of exploration of Hsp83 or Membrane transporter D1 as a drug target in *L. donovan*i. The results offer the opportunity to characterize the chemical sensitivity of the parasitic chaperone and Membrane transporter D1 against our library of Betulin derivatives *L. donovani* inhibitors with biophysical and biochemical techniques.

## Conclusion

In this study, Recursive partitioning (both ST and BF) methods were firstly used to develop classification models for the inhibitory activity of 58 betulin derivatives in vitro against *L. donovani* amastigotes. These models can be used to screen a large compound library for facilitating the discovery of the novel lead compounds. Most relevant molecular features of betulin derivative inhibition were identified. These features provide an excellent analytical perspective to explain the similarities and differences between betulin derivative inhibitors and non-inhibitors. The potential targets of these compounds were determined through in silico target fishing, which combines 3D structure-based pharmacophore searching and network pharmacology analysis. Using this strategy, we inferred links between most active compounds and Leishmaniasis disease through molecular targets and keys signaling pathways. Further studies need to validate identified targets and to test the effects of betulin derivatives on identified pathways and their interactions (Additional file 4: Fig. S2, Additional file 5).

## Additional files

**Additional file 1: Table S1**. Bagged forest confusion matrix.

**Additional file 2: Table S2**. Single tree confusion matrix.

**Additional file 3: Fig. S1**. Full pharmacological network of Leishmania donovani Betulin derivatives inhibitors. Betulin derivatives inhibitors, pharmacophore, targets and biopathway with a red to gray gradient scale.

**Additional file 4: Fig. S2**. Superimposition of potential protein target structures.

**Additional file 5.** Zip file with all potential protein target structures protein data bank files.
