# Supplementary material for: Predictive classification models and targets identification for betulin derivatives as Leishmania donovani inhibitors
Source: J Cheminform. 2018 Aug 17;10:40. doi: 10.1186/s13321-018-0291-x (PMC6097978; doi:10.1186/s13321-018-0291-x)
Supplement: Supplementary file 1 — Additional file 1: Table S1. Bagged forest confusion matrix. [file 13321_2018_291_MOESM1_ESM.docx]

**Sup 1. Bagged forest confusion matrix**

| Dataset | Split method |  | Confusion matrix | | | Sensitivity | Specificity |
| --- | --- | --- | --- | --- | --- | --- | --- |
| 2 Class from 3 | Diversity | In-bag training data results for 10 trees |  | 1 | 3 | 1 | 0.87 |
|  |  |  | 1 | 11 | 2 |  |  |
|  |  |  | 3 | 0 | 13 |  |  |
|  |  | Out-of-bag training data results for 10 trees |  | 1 | 3 | 0.58 | 0.54 |
|  |  |  | 1 | 7 | 6 |  |  |
|  |  |  | 3 | 5 | 7 |  |  |
|  |  | External test set |  | 1 | 3 | 0.89 | 0.75 |
|  |  |  | 1 | 8 | 1 |  |  |
|  |  |  | 3 | 1 | 3 |  |  |
| 2 Class from 3 | RC | In-bag training data results for 10 trees |  | 1 | 3 | 0.95 | 0.80 |
|  |  |  | 1 | 18 | 2 |  |  |
|  |  |  | 3 | 1 | 8 |  |  |
|  |  | Out-of-bag training data results for 10 trees |  | 1 | 3 | 0.80 | 0.63 |
|  |  |  | 1 | 16 | 3 |  |  |
|  |  |  | 3 | 4 | 5 |  |  |
|  |  | External test set |  | 1 | 3 | 0.25 | 1 |
