# Supplementary material for: Predictive classification models and targets identification for betulin derivatives as Leishmania donovani inhibitors
Source: J Cheminform. 2018 Aug 17;10:40. doi: 10.1186/s13321-018-0291-x (PMC6097978; doi:10.1186/s13321-018-0291-x)
Supplement: Supplementary file 2 — Additional file 2: Table S2. Single tree confusion matrix. [file 13321_2018_291_MOESM2_ESM.docx]

**Sup 2. Single tree confusion matrix**

| Dataset | Split method |  | Confusion matrix | | | Sensitivity | Specificity |
| --- | --- | --- | --- | --- | --- | --- | --- |
| 2 Class from 3 | Diversity | Tree 1: 4 leaves |  | 1 | 3 | 0.80 | 0.91 |
|  |  |  | 1 | 12 | 1 |  |  |
|  |  |  | 3 | 3 | 10 |  |  |
|  |  | External test set |  | 1 | 3 | 0.80 | 0.67 |
|  |  |  | 1 | 8 | 1 |  |  |
|  |  |  | 3 | 2 | 2 |  |  |
| 2 Class | RC | Tree 1: 6 leaves |  | 1 | 3 | 0.88 | 0.78 |
|  |  |  | 1 | 21 | 4 |  |  |
|  |  |  | 3 | 3 | 14 |  |  |
|  |  | External test set |  | 1 | 3 | 0.60 | 1 |
|  |  |  | 1 | 6 | 0 |  |  |
|  |  |  | 3 | 4 | 6 |  |  |
