# Supplementary material for: Predictive classification models and targets identification for betulin derivatives as Leishmania donovani inhibitors
Source: J Cheminform. 2018 Aug 17;10:40. doi: 10.1186/s13321-018-0291-x (PMC6097978; doi:10.1186/s13321-018-0291-x)

**Sup 3. Full pharmacological network of Leishmania donovani Betulin derivatives inhibitors. Betulin derivatives inhibitors, pharmacophore, targets and biopathway with** **a red to gray gradient scale.**


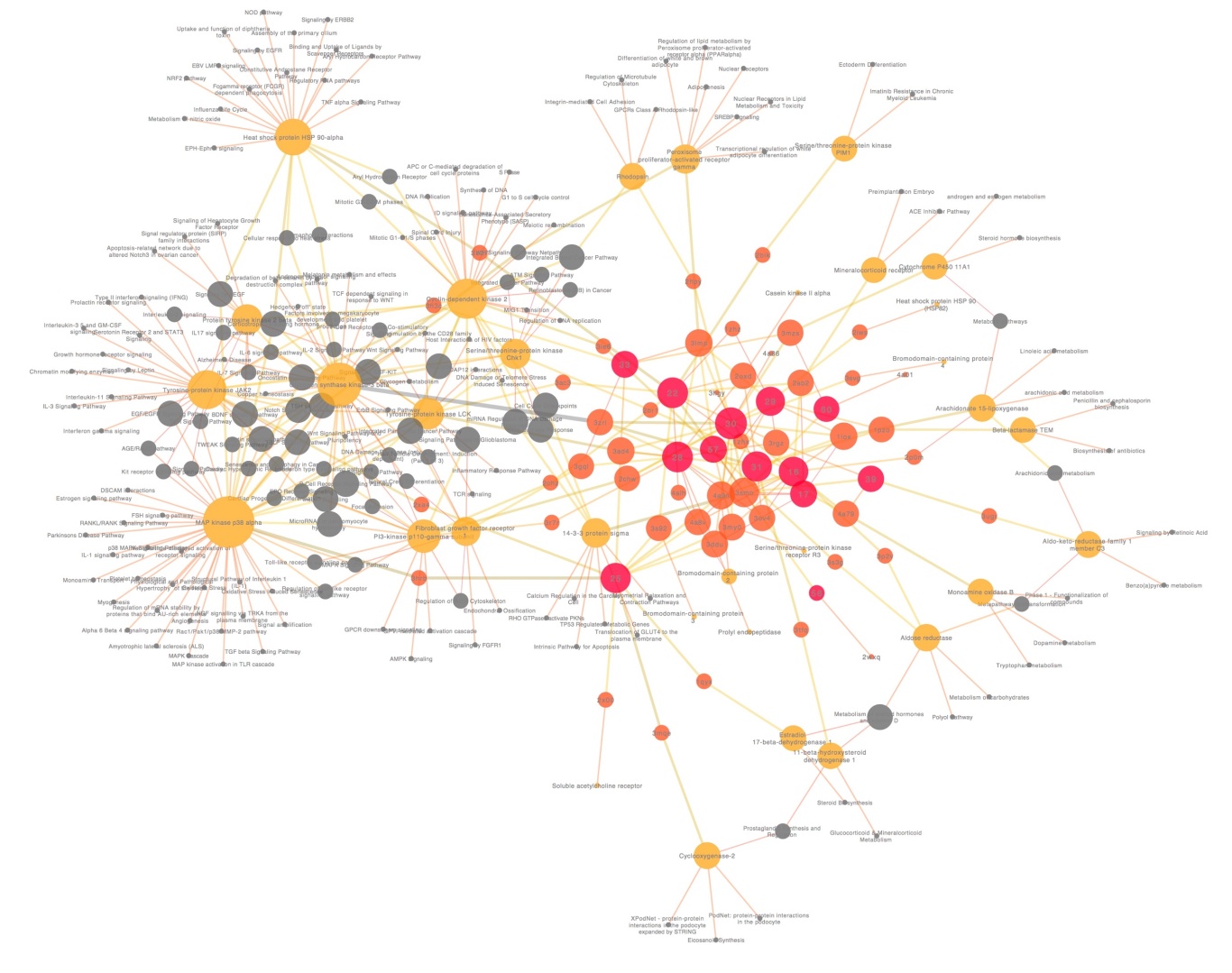

Supplement: Supplementary file 3 — Additional file 3: Fig. S1. Full pharmacological network of Leishmania donovani Betulin derivatives inhibitors. Betulin derivatives inhibitors, pharmacophore, targets and biopathway with a red to gray gradient scale. [file 13321_2018_291_MOESM3_ESM.docx]
