# Supplementary figures and images for: Predictive classification models and targets identification for betulin derivatives as Leishmania donovani inhibitors
Source: J Cheminform. 2018 Aug 17;10:40. doi: 10.1186/s13321-018-0291-x (PMC6097978; doi:10.1186/s13321-018-0291-x)

**Sup 4. Superimposition of potential protein target structures**


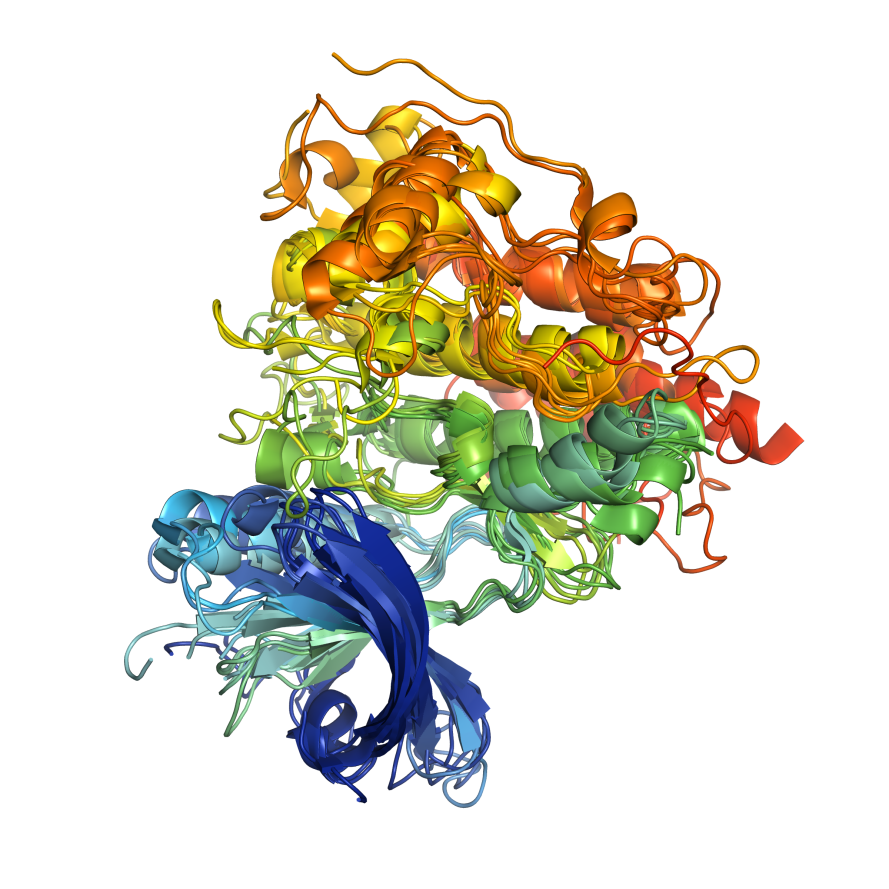

Supplement: Supplementary file 4 — Additional file 4: Fig. S2. Superimposition of potential protein target structures. [file 13321_2018_291_MOESM4_ESM.docx]
